# How modularity and heterotrophy complicate the understanding of the causes of thermal performance curves: the case of feeding rate in a filter-feeding animal

**DOI:** 10.1242/jeb.247776

**Published:** 2024-06-26

**Authors:** Jackson A. Powell, Scott C. Burgess

**Affiliations:** Department of Biological Science, Florida State University, 319 Stadium Drive, Tallahassee, FL 32306-4296, USA

**Keywords:** Climate change, Thermal tolerance, Modular organisms, Marine, Feeding systems, Indirect effects

## Abstract

Warming global temperatures have consequences for biological rates. Feeding rates reflect the intake of energy that fuels survival, growth and reproduction. However, temperature can also affect food abundance and quality, as well as feeding behavior, which all affect feeding rate, making it challenging to understand the pathways by which temperature affects the intake of energy. Therefore, we experimentally assessed how clearance rate varied across a thermal gradient in a filter-feeding colonial marine invertebrate (the bryozoan *Bugula neritina*). We also assessed how temperature affects phytoplankton as a food source, and zooid states within a colony that affect energy budgets and feeding behavior. Clearance rate increased linearly from 18°C to 32°C, a temperature range that the population experiences most of the year. However, temperature increased algal cell size, and decreased the proportion of feeding zooids, suggesting indirect effects of temperature on clearance rates. Temperature increased polypide regression, possibly as a stress response because satiation occurred quicker, or because phytoplankton quality declined. Temperature had a greater effect on clearance rate per feeding zooid than it did per total zooids. Together, these results suggest that the effect of temperature on clearance rate at the colony level is not just the outcome of individual zooids feeding more in direct response to temperature but also emerges from temperature increasing polypide regression and the remaining zooids increasing their feeding rates in response. Our study highlights some of the challenges for understanding why temperature affects feeding rates, especially for understudied, yet ecologically important, marine colonial organisms.

## Teaser

**Summary:** The effect of temperature on individual feeding rate can emerge from the indirect effects on colony composition in modular animals and phytoplankton food quality.

## INTRODUCTION

Amongst the numerous consequences of climate change, rising temperature is often considered to be one of the most concerning (Dillon et al., 2010; Easterling et al., 2000). The ecological consequences anticipated by a warmer climate include decreasing body mass (Atkinson, 1994; Atkinson et al., 2003; Smith et al., 1998), shorter generation times (Ahn et al., 2016; Altermatt, 2010; Parmesan and Yohe, 2003), shifts in geographic ranges and range limits (Parmesan and Yohe, 2003; Pecl et al., 2017; Pinsky et al., 2013; Sunday et al., 2012), and a myriad of other physiological and behavioral effects (Huey et al., 2012). A common way to measure thermal sensitivity is to assess the shape of a thermal performance curve, which describes the relationship between temperature and organism performance (Huey and Stevenson, 1979). Traits chosen to assess performance should be those necessary for an organism to survive and reproduce. As such, many studies employing thermal performance curves have used important physiological traits such as respiration, growth rates, photosynthesis and traits affecting other metabolic processes (Jonsson et al., 2001; Padfield et al., 2016, 2017; Silbiger et al., 2019).

A challenge of attempting to estimate the thermal sensitivity of performance traits is that the effects of temperature can affect performance directly, or indirectly through interactions with other organisms (Kharouba and Yang, 2021; Murrell and Barton, 2017). The performance of virtually all organisms is influenced to some degree by interactions with other organisms. The interactions themselves can be altered by warming temperatures (Anderson et al., 2001; Gilman, 2017; Lemoine and Burkepile, 2012; Rall et al., 2012; Sanford, 2002). For example, thermally sensitive species are usually more impacted by temperature-dependent species interactions than are less thermally sensitive species (Diamond et al., 2017). Furthermore, the effects of warming on a prey species alone can result in detrimental effects on a predator species that warming has neutral or positive direct effects upon (Kharouba and Yang, 2021). Therefore, thermal performance curves are essentially a correlation between performance and temperature, where it is often assumed that temperature directly affects performance. However, it has been challenging to understand how direct and indirect effects generate thermal performance curves in natural settings. Assuming that the relationship between temperature and performance is solely driven by the direct effects of temperature could be erroneous if the interactions between temperature, the focal organism's traits and the traits of other organisms with which it interacts are not taken into account (Anderson et al., 2001; Gilman, 2017; Kharouba and Yang, 2021; Lemoine and Burkepile, 2012).

An important component of organism performance is feeding. In marine environments, food such as phytoplankton and zooplankton is suspended in the water column, and many animals acquire such food items through actively filtering water across feeding structures via the generation of currents (Denny, 1990). Temperature can directly affect feeding rates because of temperature-dependent metabolic requirements and stress responses (Huey and Stevenson, 1979; Menon, 1974; Riisgård and Manríquez, 1997; Sinclair et al., 2016). However, temperature can indirectly affect feeding rates by changing the amount and nutritional quality of food items. For example, warming temperatures affect the quality of phytoplankton as a food source by altering fatty acid content, nitrogen:phosphorus ratios and carbon:phosphorus ratios, which affect nutritional demand, growth efficiency and reproduction in consumers (Bi et al., 2017; D'Souza and Kelly, 2000; Fuschino et al., 2011; Hessen et al., 2017; Kharouba and Yang, 2021; Kiørboe, 1989; Klein Breteler et al., 2005; Rothhaupt, 1995; Sikora et al., 2014; Tseng et al., 2021). Furthermore, increased temperature decreases water viscosity, which can affect both the consumer's ability to generate currents through small feeding structures such as setae or cilia to capture prey, and the prey's ability to avoid consumption (Jørgensen, 1983; Miyake and Koizumi, 1948; Peleg, 2018; Podolsky, 1994; Tyrell et al., 2020). Marine organisms that feed through capturing particles suspended in the water column may therefore be affected by temperature indirectly through its effects on the physiology and nutritional quality of their food items, and the physical changes that it has on their environment.

The response of a trait's performance to temperature may also depend on the body plan of the animal. Many aquatic and marine taxa have a modular body plan in which an individual is a colony composed of multiple clonal modules (e.g. polyps in corals, or zooids in bryozoans and ascidians) with varying degrees of inter-dependence and function. Modular body plans result in different organismal interactions with the environment compared with solitary animals (Burgess et al., 2017; Echer et al., 2014; Johnston et al., 1981; Konsens et al., 1991). Specifically, the feeding rates of modular organisms may respond to environmental changes in different ways to those of unitary animals because of differences in metabolic scaling with size and the ability of colonies to adjust the number of modules capable of feeding (Barneche et al., 2017; Burgess et al., 2017). The number of zooids in a colony relates to the number of potential feeding modules (autozooids) that can acquire food. Food supply depends on the number of food items per unit volume of water and current speed, which then influence feeding rates (Best and Thorpe, 1983, 1986; Bullivant, 1968a; Hunter and Hughes, 1993; Riisgård, 1991; Riisgard and Randløv, 1981). In the case of bryozoans, colonies can also have some fraction of zooids in non-feeding states, where the feeding and muscular structures within individual zooids (the polypide) temporarily degenerate into a cluster of undifferentiated cells (‘brown bodies’) through a process called ‘polypide regression’ (Bayer et al., 1994; Bobin, 1964; Nekliudova et al., 2019; Palumbi and Jackson, 1983; Ries, 1936). The brown body of a regressed zooid is not dead but has reduced metabolic needs. The proportion and distribution of regressed zooids can also affect cilia-generated water flow through the colony from feeding zooids (McKinney et al., 1986). Therefore, any investigation of feeding rates in modular organisms should consider the responses of food items to temperature, the number of zooids that are available for feeding within a colony, and the factors that could interact with temperature to influence feeding rates beyond temperature on its own.

The primary goal of this study was to estimate the relationship between temperature and feeding rate in a sessile filter feeder (the marine bryozoan *Bugula neritina*). We also sought to determine the indirect effects of temperature on feeding rate through its effects on phytoplankton cell concentration, size and internal complexity, as well as zooid states related to feeding and polypide regression. We predicted that feeding rates would increase with temperature, at least until a thermal optimum was reached (Huey and Stevenson, 1979). We expected algal food to decrease in quality with increasing temperature, and that low-quality food could increase feeding rate if more algal cells were needed to meet nutritional requirements or decrease feeding rate if colonies rejected suboptimal algal cells. Finally, we expected that the proportion of zooids with regressed polypides would increase with temperature, and result in temperature affecting feeding rate differentially at the colony level versus zooid level.

## MATERIALS AND METHODS

### Study species

*Bugula neritina* (Linnaeus 1758) is an arborescent bryozoan in which individual colonies grow through the addition of clonal zooids from a sexually produced larva after it attaches to hard substrate. After settling, larvae metamorphose into the first zooid (ancestrula) of the colony. The first zooid then produces clonal zooids to increase the size of the colony. Zooids within a colony are connected with each other structurally by the cystid, and physiologically by the funicular system. Each zooid contains the necessary organs for feeding, growth and reproduction. Zooids feed using lophophores, which form a ring of hollow tentacles surrounding the mouth and are lined with cilia that create currents that pull food particles from the water column into their mouths (Bullivant, 1968b; Okamura, 1990; Pratt, 2005). Like other bryozoans, the behavior and function of individual zooids within the colony can vary, and polypides regularly regress into brown bodies depending on the environmental conditions (Crowell, 1953; Nekliudova et al., 2019).

### Sample collection

*Bugula neritina* for the parental generation were collected in May 2023 from shallow seagrass habitats at Dog Island (29°49′42.0″N 84°34′50.9″W) in the Gulf of Mexico, roughly 5.5 km offshore from the Florida State University Coastal and Marine Lab (FSUCML), FL, USA. Over the last 18 years, field water temperatures in May have averaged 25.84±1.75°C (mean±s.d.) (https://marinelab.fsu.edu/water-conditions/, accessed 2 February 2024). The colonies collected were primarily attached to seagrass blades of either *Thalassia testudinum* or *Halodule wrightii* at 0.5–1.5 m in depth. The water temperature on the day of collection was 27.7°C. To reduce prior environmental effects and differences in colony size and age, we performed the experiment with colonies that were reared under controlled laboratory conditions from larval release. To induce larval release, colonies were placed in a cooler in complete darkness for 2 days under ambient lab conditions (∼21.5±0.5°C), after which they were placed into individual bowls of ∼250 ml filtered seawater (FSW) collected from the FSUCML and exposed to artificial light. The larvae then settled and attached to roughened and biofilm-coated acetate sheets that were floated on the water in each dish. Two to four offspring (settlers) from each mother colony were cut out of the acetate sheet and were attached to polystyrene weighing boats that allowed the settlers to suspend facing downwards in bowls filled with 250 ml of FSW and covered with lids to reduce evaporation. Only siblings that shared a mother colony shared a bowl to grow in, resulting in 2–4 sibling settlers per bowl during the growth phase. Every 2–3 days, the water was changed in each bowl and the colonies were fed with a lab-cultured cryptophyte alga, *Rhodomonas salina*, at approximately 100,000 cells ml^−1^. Cell cultures were centrifuged to remove L1 growth media before being added to the bowls. The colonies grew under ambient lab conditions (∼21.5±0.5°C) until the beginning of the experiment. Across seawater collections throughout the experiment, salinity ranged from only 27‰ to 31‰, and all colonies were given the same FSW at each water change.

### Experimental methods

After growing for 22 days (batch 1) or 25 days (batch 2), 1–2 colonies per mother (from the parental generation) were collected and randomly assigned to eight temperatures ranging from 18 to 32°C. During this time (June), mean (±s.d.) water temperature at our collection site is 28.46±1.24°C (https://marinelab.fsu.edu/water-conditions/, accessed 2 February 2024). At each target temperature (18, 20, 22, 24, 26, 28, 30 and 32°C), there was a water bath consisting of a plastic storage container filled with water which housed six covered plastic bowls containing 250 ml of FSW with a salinity of 28.3‰. Four of the bowls housed one *B. neritina* colony, each from a different mother; the remaining two bowls were without a *B. neritina* colony, and were used to estimate changes to algal concentration in the absence of colonies. All water baths were placed in a Percival incubator set to 18°C on a 13 h:11 h light:dark cycle. Each water bath (except for those assigned to 18°C) had a 50 W Hydor or Freesea aquarium heater placed into it that was attached to an Aqua Logic NEMA 4X (TR115SN) digital temperature controller. Temperature controllers were programmed to turn aquarium heaters on when water bath temperatures dropped below 0.5°C of their target temperature and to turn off when the targeted temperature was reached. HOBO Tidbit v2 temperature loggers in each water bath recorded temperature every 15 min to estimate the actual temperature of the water bath. The realized temperature experienced by the experimental colonies was calculated as the median temperature over time, starting when the colonies were first added to the water baths and ending at the close of the experiment.

Experimental colonies were fed with algal cells at approximately 30,000–46,000 cells ml^−1^, which avoided causing flocculation and congestion of cells around zooid mouths (Hunter and Hughes, 1993). When colonies were initially placed into their dishes, the heaters were turned on and colonies were allowed 1 day of acclimation. After 1 day, the water and algae were replaced in every bowl and 0.5–1.0 ml aliquots of water were collected via pipette from each bowl to estimate starting cell concentration, cell size and internal complexity of algae. Pipettes were used to thoroughly mix the contents of the water prior to the sample being taken. After 48 h, another 0.5–1.0 ml aliquot was taken from every bowl to estimate the final cell concentration, cell size and internal complexity of algae. Each *B. neritina* colony was preserved in 95% ethanol after the 48 h aliquot was collected. The 48 h duration of the experiment was similar to previous studies (Bullivant, 1968a), and integrates the effects of any temporary fluctuations in ciliary activity and feeding behavior.

### Cytometric analysis

Cell concentration, cell size and cell internal complexity of algae were estimated using a CytoFLEX Flow Cytometer (Beckman-Coulter) with the Cytexpert software program. Sample names were obscured during the cytometric analysis to ensure that we were blind to each sample's identity. Events detected by the flow cytometer were gated such that only particles that (1) were within the known size range and (2) were in the known absorption area range (400–560 nm) of cultured *R. salina* were considered to be live algae. Particles that were too small, outside the gated fluorescence range of chlorophyll-A, or both, were treated as non-algal particles and were excluded from any analyses. The gated algal populations were the dominant event in the samples tested.

Algal concentrations were estimated from the number of median particles per microliter within the gated algal population. Forward scatter area (FSC-A) and side scatter area (SSC-A) were estimated from median irradiances for the gated algal population. FSC-A is commonly used as a proxy for cell size because it is primarily due to light diffraction around the cell, where a greater intensity in FSC-A indicates cells of greater diameter. SSC-A provides information about the internal complexity (or ‘granularity’) of cells because it measures light refracted or reflected by the cell (Chioccioli et al., 2014; Mullaney and Dean, 1970; Nam et al., 2018; Samadani et al., 2018). A greater intensity in SSC-A indicates cells with more intracellular structures that reflect or refract light, such as cytoplasmic granules and nuclei. Both metrics are reported as cell size and composition of biomolecules within cells could indicate quality as food items.

As laser wavelength, sheath fluid and biological characteristics of the samples can all influence FSC-A and SSC-A (Dubelaar and Jonker, 2000), these measurements are considered to be proportional to cell size and cell internal complexity within a given study rather than direct measurements of these characteristics. For batch 1, 0.240 ml min^−1^ were analyzed for each aliquot, and for batch 2, 0.030 ml min^−1^ were analyzed for each aliquot (flow speed did not affect our estimates).

### Clearance rate

To estimate clearance rate, which is defined as the volume of water cleared of suspended particles per unit of time (Riisgård, 2001), we had to account for changes over time (*t*) in cell concentration (*A*) due to the birth rate (*b*) and death rate (*d*) of live phytoplankton cells that occurred independently of *B. neritina* feeding, in addition to changes due to *B. neritina* feeding rate (*c*), which also depends on the number of feeding zooids (*Z*). The change in the number of cells over time, d*A*/d*t*, can be represented as:
(1)

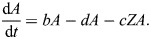
Therefore, the cell concentration at any time *t* is:
(2)

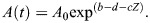
We estimated the average rate of change in cell concentration for each of the two bowls containing algae only at each temperature, which we will denote as *b*−*d*=*r*, and for each of the four bowls containing algae and *B. neritina* at each temperature, which we will denote as *r*−*cZ*, between day 0 (initial) and day 2 (final) as:
(3)

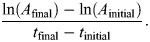
We then took the average value of *r* and *r*−*cZ* at each temperature, 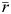 and 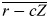, respectively. Therefore, the difference in the rate of change in algal concentration between the bowls with algae only and the bowls with algae and *B. neritina*, 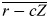
−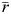
=*F*, estimates the rate of change in cell concentration due to *B. neritina* feeding at each temperature. The estimated clearance rate per colony (each bowl contained *N*=1 colony and a volume, *V*, of 250 ml) at each temperature was then defined as:
(4)

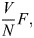
in units of ml day^−1^ colony^−1^. The clearance rate per zooid or per feeding zooid (*Z*) was defined as:
(5)

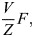
and is in units of ml day^−1^ zooid^−1^ (Riisgård, 2001).

### Zooid states

The number of zooids in each of three states was counted in preserved colonies of *B. neritina* under a light microscope. The three zooid states were: (1) zooids capable of feeding (possessing a visible gut and lophophore tentacles), (2) regressed zooids (visible brown bodies or tissue lacking lophophores), and (3) dead zooids (cystids that were visibly empty).

### Statistical analyses

The relationships between realized temperature and the change in algal concentration, clearance rate per colony, clearance rate per zooid and clearance rate per feeding zooid were estimated using Gaussian linear models. The relationship between realized temperature and the total number of zooids (sum of the number of zooids in all three states) was estimated using negative binomial generalized linear models. The relationship between realized temperature and the proportion of zooids in a given state was estimated using binomial generalized linear models. For all response variables, batch was included as a fixed effect, and we fitted realized temperature as a first, second, third or fourth order polynomial. We used Akaike's information criterion for small sample sizes (AICc) to determine which order of polynomial best fitted the data (with batch fitted as an interactive effect). When multiple models fitted the data equally well (ΔAICc<2), we chose the model with the lowest degree of polynomial. Once the best model of curvature was found, we used χ^2^ likelihood ratio tests to determine whether there were interactive or additive effects of batch, as well as whether the slope parameters describing the relationship between realized temperature and the response were significantly different from zero. All models were fitted using the package ‘glmmTMB’ (version 1.1.7) in R (version 4.3.1).

## RESULTS

### Thermal gradient

The realized temperature in each of the eight water baths was within 1.10°C of the target temperature, indicating that temperatures were adequately maintained. Temperature fluctuations for a specific target temperature exhibited coefficients of variation ranging from 0.819% to 2.856%, which meant that fluctuations over time were much smaller than the 2°C difference between target temperatures.

### Temperature effects on the concentration and physical characteristics of algae

The average cell concentration at the start of the experiment in batch 1 was 31,306 cells ml^−1^ (95% confidence interval, CI: 30,130–32,481 cells ml^−1^), and in batch 2 was 43,438 cells ml^−1^ (95% CI: 41,118–45,757 cells ml^−1^).

**Fig. 1.**
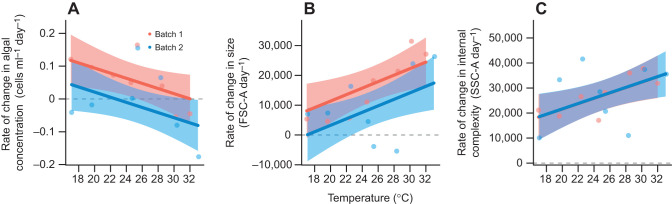
**The effects of temperature on the concentration and physical characteristics of algae.** (A) Rate of change in algal concentration, *r*. (B) Rate of change in algal cell size (measured as forward scatter area, FSC-A). (C) Rate of change in algal cell internal complexity (measured as side scatter area, SSC-A). Data are from two experimental runs (batches). Data points at each temperature represent the mean of two bowls without *Bulgula neritina* present. The red (batch 1, *n*=8) and blue (batch 2, *n*=8) bands represent 95% confidence intervals (CI) estimated from linear (polynomial) models.

In the absence of *B. neritina*, there was no evidence for a curvilinear relationship between realized temperature and the rate of change in cell concentration (Table 1). The rate of change in cell concentration declined by 0.008 cells ml^−1^ day^−1^ (95% CI: 0.001–0.015 cells ml^−1^ day^−1^) for every 1°C increase in realized temperature (χ^2^=3.939, d.f.=1, *P*=0.047; Fig. 1A). There was no interaction between realized temperature and batch (χ^2^=0.717, d.f.=1, *P*=0.397), nor an additive effect of batch (χ^2^=3.595, d.f.=1, *P*=0.058). The proxies for cell size (FSC-A) and internal complexity (SSC-A) both increased over the course of the experiment, and the rate of change in FSC-A day^−1^ increased with temperature (χ^2^=7.753, d.f.=1, *P*=0.005) while the rate of change in SSC-A day^−1^ was statistically independent of temperature (χ^2^=3.771, d.f.=1, *P*=0.052; Fig. 1B,C). The rate of change in FSC-A increased by 1089.632 FSC-A day^−1^ (95% CI: 256.507–1922.756 FSC-A day^−1^) for every 1°C increase in realized temperature, revealing that colonies in warmer temperatures would be consuming cells with greater diameters than colonies in lower temperatures. The rate of change in FSC-A day^−1^ in batch 2 was 8117.829 FSC-A day^−1^ (95% CI: −11.005–16246.663 FSC-A day^−1^) lower than in batch 1.

**Table 1.**
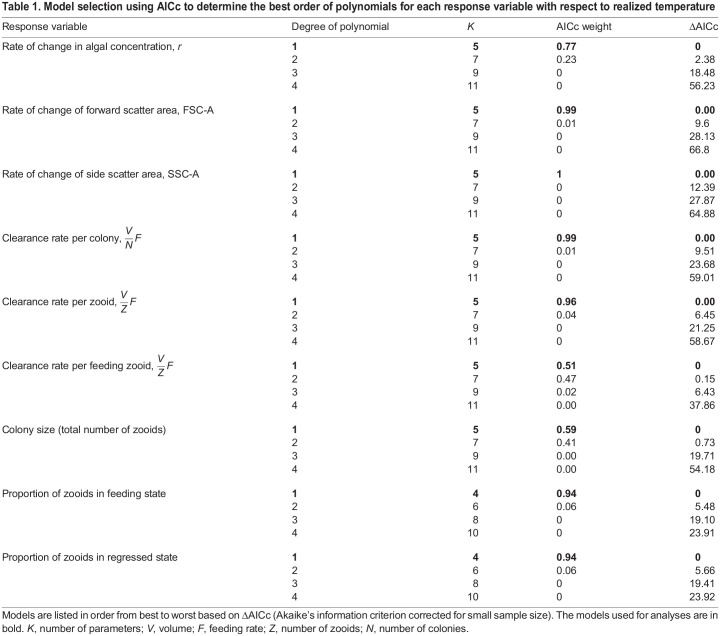
Model selection using AICc to determine the best order of polynomials for each response variable with respect to realized temperature

### Temperature effects on clearance rates

**Fig. 2.**
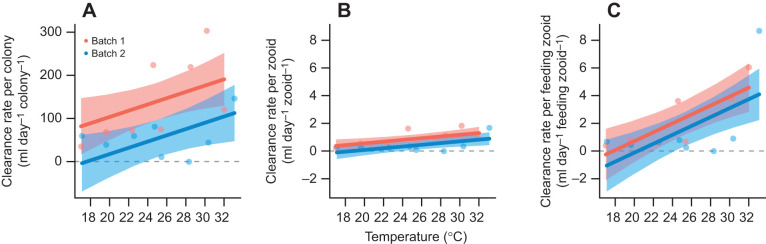
**The effects of temperature on algal clearance rate in *B. neritina*.** (A) Colony-level clearance rate. (B) Clearance rate per zooid. (C) Clearance rate per feeding zooid. Data are from two experimental runs (batches). The red (batch 1) and blue (batch 2) data points represent the mean of four replicate clearance rate estimates within each temperature. The red (batch 1, *n*=8) and blue (batch 2, *n*=8) bands represent 95% CI estimated from linear (polynomial) models.

**Fig. 3.**
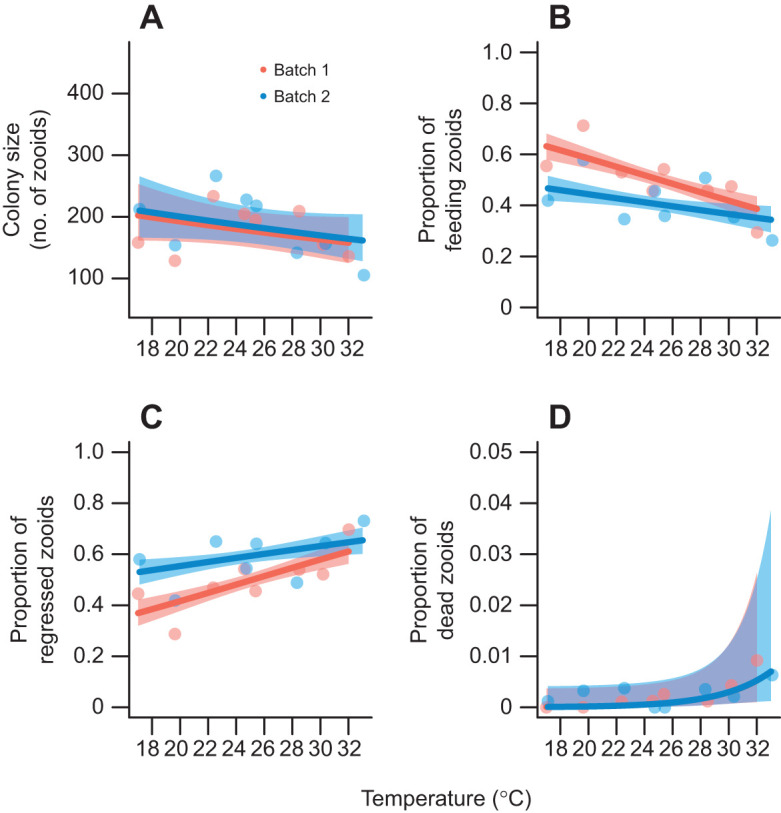
**The effects of temperature on feeding capacity of *B. neritina* zooids.** (A) Colony size (total number of zooids). (B) The proportion of zooids capable of feeding. (C) The proportion of regressed zooids. (D) The proportion of dead zooids. Data are from two experimental runs (batches). Note the change in *y*-axis scale in D compared with B and C for easier visualization. The red (batch 1, *n*=8) and blue (batch 2, *n*=8) bands represent 95% CI estimated from linear (polynomial) models.

There was no evidence of curvilinearity in the relationship between clearance rate and temperature, regardless of whether clearance rate was per colony, per zooid or per feeding zooid (Table 1). Colony-level clearance rate increased with temperature at a rate of 7.265 ml day^−1^ colony^−1^ (95% CI: 1.232–13.298 ml day^−1^ colony^−1^) for each 1°C increase (Fig. 2A) and the rate was statistically different from zero (χ^2^=4.779, d.f.=1, *P*=0.029). Colonies from batch 1 fed at a rate of 86.124 ml day^−1^ colony^−1^ (95% CI: 26.485–145.764 ml day^−1^ colony^−1^) higher than colonies from batch 2 (χ^2^=6.495, d.f.=1, *P*=0.011) because they had a higher proportion of feeding zooids rather than having more zooids (Fig. 3A,B).

Clearance rate per zooid increased by 0.062 ml day^−1^ zooid^−1^ (95% CI: 0.019–0.105 ml day^−1^ zooid^−1^) for each 1°C increase (χ^2^=6.503, d.f.=1, *P*=0.011; Fig. 2A). However, not all zooids were in the feeding state, and clearance rate per feeding zooid increased more quickly, by 0.323 ml day^−1^ zooid^−1^ (95% CI: 0.150–0.494 ml day^−1^ zooid^−1^) for each 1°C increase (χ^2^=9.857, d.f.=1, *P*=0.002). Colonies from batch 1 had a clearance rate of 0.476 ml day^−1^ zooid^−1^ (95% CI: 0.048–0.904 ml day^−1^ zooid^−1^) higher than those from batch 2 (χ^2^=4.156, d.f.=1, *P*=0.041; Fig. 2B), but not per feeding zooid (χ^2^=0.894, d.f.=1, *P*=0.345; Fig. 2C).

### Temperature effects on feeding capacity (zooid states)

On average, colonies in the experiment contained 184 zooids (95% CI: 162–205 zooids). Colony size (total number of zooids) did not differ across temperatures (χ^2^=2.186, d.f.=1, *P*=0.139) or between batches (χ^2^=0.12, d.f.=1, *P*=0.729; Fig. 3A). However, the proportion of feeding zooids decreased linearly with increasing temperature, though the proportion of feeding zooids decreased more rapidly in batch 1 colonies than in batch 2 colonies (χ^2^=4.305, d.f.=1, *P*=0.038; Fig. 3B). Conversely, the proportion of regressed zooids increased linearly with temperature, though at different rates in each batch (χ^2^=3.99, d.f.=1, *P*=0.046; Fig. 3C). Curvilinearity for the relationship between the proportion of dead zooids and temperature could not be assessed because models above first order polynomials failed to converge. The proportion of dead zooids per colony remained low across all temperatures, though it increased slightly at the higher temperatures above 28°C (χ^2^=4.942, d.f.=1, *P*=0.026; Fig. 3D).

## DISCUSSION

**Fig. 4.**
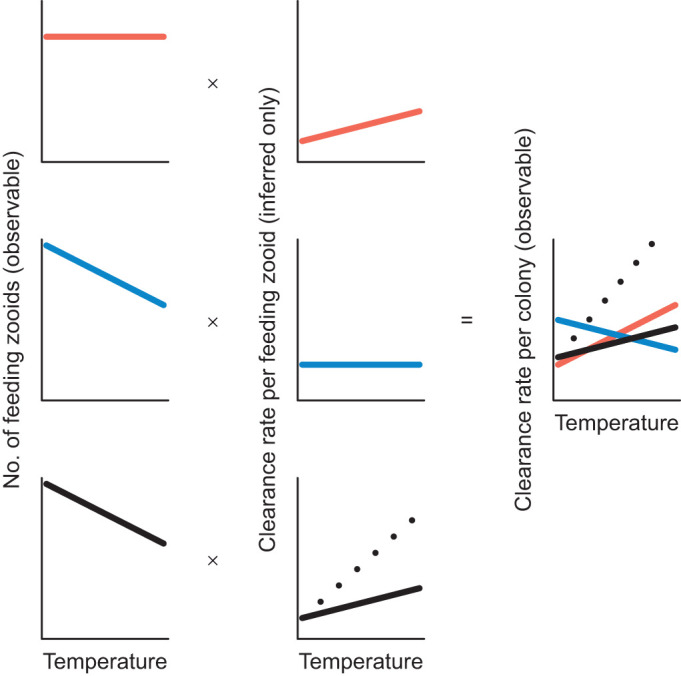
**Hypothetical scenarios depicting how clearance rate per colony (which is observable) can emerge from the number of feeding zooids in the colony (which is also observable) and the clearance rate per feeding zooid (which is not directly observable).** An increase in clearance rate per colony with increased temperature can arise simply by an increase in clearance rate per feeding zooid (red line) and requires individual zooids to feed more (blue line). A decreasing number of feeding zooids with increasing temperature would then decrease the effect of temperature at the colony level (blue and solid black lines). However, zooids increase their feeding rate in response to there being fewer feeding zooids, and more regressed zooids in the colony would then increase the effect of temperature at the colony level (dotted black line).

Feeding rates reflect an organism's ability to acquire energy to meet metabolic demands that fuels survival, growth and reproduction, so it is important to understand the thermal sensitivity of feeding rate, especially in the context of global warming (D'Souza and Kelly, 2000; Kharouba and Yang, 2021; Kiørboe, 1989; Klein Breteler et al., 2005; Rothhaupt, 1995; Sikora et al., 2014; Tseng et al., 2021). Therefore, our goal was to understand the effect of temperature on feeding rate in a sessile filter feeder (*Bugula neritina*), and to better understand how feeding rates could be influenced by the effects of temperature on food abundance (cell concentration) and composition (proxies for cell size and internal complexity), as well as directly on feeding capacity (zooid states). Our experimental approach used live phytoplankton and accounted for the effect of temperature on the growth and death of cells that occurred independently of bryozoan feeding. Clearance rate increased linearly from 18°C to 32°C, which are temperatures experienced over approximately 6 months of the year (May to October) in the field (Keough and Chernoff, 1987). However, the proportion of zooids in each colony that were capable of feeding decreased with temperature, making it challenging to understand the pathways by which temperature affects feeding at the colony level (Fig. 4).

Fig. 4 conceptualizes how clearance rate of the colony (which is an observable quantity) can emerge from the number of feeding zooids in the colony (which is also an observable quantity) and the clearance rate per feeding zooid. The latter is not directly observable but, in addition to the number of mouths, is key to understanding what emerges at the colony level. If temperature had no effect on the proportion of zooids in each colony that were capable of feeding, or had we not measured it, then we would infer that the observed effect of temperature on clearance rate per colony simply reflects the effect of temperature on clearance rate per zooid (i.e. the slopes in Fig. 3B and C would be similar). Under this scenario, the increased clearance rate at the colony level would simply reflect the effect of temperature causing zooids to feed more actively to meet metabolic demands (Fig. 4, red lines). If instead there were fewer feeding zooids at higher temperatures compared with lower temperatures, as we observed, then there would be fewer mouths, so temperature would decrease clearance rate at the colony level (Fig. 4, blue and solid black lines). Although a positive relationship between temperature and colony-level clearance rates emerges when individual zooids feed more at higher temperatures (Fig. 4), it is also possible that zooids increase their feeding rate in response to there being fewer feeding zooids and more regressed zooids in the colony. The increased feeding rate of individual zooids would then act to increase the effect of temperature on clearance rate at the colony level beyond that expected if zooids fed at the lower rate estimated when all zooids feed (Fig. 4, dotted versus solid black lines). The expected feeding rate per zooid at higher temperatures could be higher than that expected if all zooids were feeding (i.e. the slope of Fig. 3C is greater than that in 3B) to offset the effect of fewer mouths at higher temperatures. Put another way, if all zooids were feeding at the rate expected for feeding zooids only (Fig. 3C), then the effect of temperature on colony-level feeding rates would be higher than that observed. It is possible that the expected feeding rate per zooid, which we cannot measure directly, is the outcome of both the effect of temperature on feeding (e.g. due to increased metabolic demands of individual zooids) and the individual zooids feeding at faster rates than they otherwise would (i.e. if all zooids were feeding) to acquire energy from food for themselves as well as for distribution to other zooids (e.g. through pore plates; Bone and Keough, 2005, 2010) in the colony that are not feeding (i.e. regressed zooids).

Our result that clearance rate increased linearly over a certain range of temperatures aligns with previous findings (Huey and Stevenson, 1979; Menon, 1974; Riisgård and Manríquez, 1997; Sinclair et al., 2016). However, because clearance rate depends on multiple factors such as cell concentration, cell size, phytoplankton species, satiation and concentration (Bayer et al., 1994; Jørgensen, 1983; Okamura, 1990; Okamura et al., 1987; Podolsky, 1994; Riisgård, 2001; Riisgård and Larsen, 2007; Riisgård and Manríquez, 1997; Tyrell et al., 2020), which can also be affected by temperature, it has been challenging to isolate the direct and indirect effects of temperature on clearance rate. That clearance rate per feeding zooid remained the same across batches, with batch 2 having ∼37% more cells per milliliter than batch 1, also indicates that variation in algal concentration did not alter our estimates of clearance rate, an effect commonly found in other studies at similar cell concentrations (Best and Thorpe, 1983, 1986).

Several of the possible causes of polypide regression may affect how feeding responds to temperature. Some have argued that a regression–regeneration cycle is related to excretion (Calvet, 1900; Marcus, 1926), and polypides regress when their stomachs are satiated from the buildup of indigestible material in the gut of each zooid, then regenerate (Harmer, 1931; Gordon, 1975; Jebram, 1975). If warmer temperatures increase feeding rate, then zooids in warmer water could accumulate waste quicker and regress sooner than they would in cooler water, causing feeding rate at the colony level to eventually decline (Harmer, 1931; Menon, 1974; Riisgård and Manríquez, 1997), if not compensated for by the remaining zooids increasing their feeding rate. Alternatively, polypide regression has also been attributed to stressful environmental conditions (Marcus, 1926; Rey, 1927), and may be an adaptive response to tolerate environmental stress or damage (Freeman, 1964; Nekliudova et al., 2019), and to prolong lifespan (Gordon, 1977; Toth, 1969) by reducing metabolic rate. In our experiments, temperature acted mostly to transition zooids from a feeding state to a regressed state, rather than killing them, which changes both the capacity to feed and the metabolic demands of the colony. Furthermore, thermally sensitive phytoplankton prey may decrease in quality with warming temperatures, and feeding rate may increase to meet nutritional requirements, or result in rapid consumption of suboptimal food that could trigger more polypides to regress (Calvet, 1900; Gordon, 1975; Jebram, 1975; Marcus, 1926; Rey, 1927; Woollacott and Zimmer, 1975). The covariation between increased clearance rates in feeding zooids at higher temperature and increased proportion of regressed zooids at higher temperatures seen here seems most parsimonious with the hypothesis that polypides regress as a result of the buildup of indigestible material in the gut.

Further, the effects of temperature on the algal food source are another indirect pathway by which temperature can affect feeding rates. Higher temperatures were found to affect forward scatter, a proxy for cell size. Thus, temperature could have indirectly affected clearance rate if food quality affects feeding rate or polypide regression (Jebram, 1975). Cell sizes increasing in warmer temperatures went against our expectations, but we hypothesize that the increase in cell size, and internal complexity, was a consequence of nitrogen and phosphorus deprivation due to algae being removed from their growth media (Berdalet, 1991; Berdalet et al., 1994; Berges and Falkowski, 1998; Dammak et al., 2021; Harrison et al., 1990; Hu, 2013; Latasa and Berdalet, 1994; Lewitus and Caron, 1990; Rasconi et al., 2015; Yuan et al., 2018). The increase in FSC-A was greater at higher temperatures, suggesting that cells in warmer temperatures were larger than those at lower temperatures. In previous studies, *B. neritina* preferred larger particles, so feeding rate may have also increased in warmer temperatures because of cells reaching larger sizes in warmer temperatures (Okamura, 1990). The increase in SSC-A across all temperatures was likely a result of algal cell macromolecule composition changes that resulted in SSC-A values increasing. Supposed changes in macromolecule composition are outlined below.

The changes in cell size and internal composition indicate that lipid composition of algal cells may have changed. While lipid composition was not directly estimated, lipid composition has been shown to change with temperature and nutrient availability across several algal taxa, with declines in proportions of polyunsaturated fatty acids (PUFAs) in warmer temperatures and nutrient-limited environments (Ahlgren et al., 1990; Atkinson et al., 2003; Fuschino et al., 2011; Rüger and Sommer, 2012; Peter and Sommer, 2012; Sikora et al., 2014; Vu et al., 2016; but see Tseng et al., 2021). Phytoplankton with a high content of highly unsaturated fatty acids (HUFAs, a subset of PUFAs), particularly eicosapentaenoic acid and docosahexaenoic acid, are considered desirable food for marine taxa as most animals cannot synthesize HUFAs on their own despite the importance of HUFAs for cell membrane structure (Brett and Müller–Navarra, 1997; Shaikh et al., 2015; Sherratt and Mason, 2018). As a result of HUFA content declining in algae grown under warm or nitrogen- and phosphorous-deprived conditions and the effects of algae grown under such conditions on the fitness of several marine taxa, algae lacking such lipids are expected to be of lower quality (Ahlgren et al., 1990; D'Souza and Kelly, 2000; Kiørboe, 1989; Klein Breteler et al., 2005; Rothhaupt, 1995). That polypide regression has previously been shown to increase as food quality declines also suggests that temperature could have indirect effects by reducing food quality (Jebram, 1975). Feeding rates may respond to decreased food quality by increasing (Flores et al., 2014), remaining constant (Kiørboe, 1989; Rothhaupt, 1995) or decreasing (Harvey and Moore, 2017) even if the response is detrimental to fitness. Ultimately, our study highlights some of the challenges for understanding the causes of thermal performance curves under experimental conditions.

Estimates of thermal performance curves also depend on the duration of temperature exposure (Kingsolver and Woods, 2016; Sinclair et al., 2016). Our estimates of the effect of temperature on algal cells, clearance rate and zooid states integrated processes over 48 h, after a period of acclimation, which was enough time to detect effects of temperature on phytoplankton quantity and quality. It was only logistically feasible to count the number of zooids and zooid states at the end, rather than the beginning, of the experiment, but as colonies were grown from larvae and were the same age in each batch, and were the same size at the end of 48 h, we assume that the number of zooids and zooid states was similar among batches prior to being exposed to temperature. Therefore, the patterns of zooid regression and clearance rate emerged during the 48 h exposure. However, only measuring the zooid states after 48 h also raises the possibility for feeding that took place early after the exposure to temperature to be accomplished with a greater number of feeding zooids than what were measured at the end of the experiment, skewing our measures of per zooid clearance rate, but not necessarily the effect of temperature on clearance rate. Studies measuring clearance rate over several minutes still find increased rates at higher temperatures, even if the overall clearance rate is higher (Hunter and Hughes, 1993; Riisgård, 2001).

This study highlights the challenges that go into predicting responses to climate change. Even within a ‘simple’ scenario in which only two species are studied, the way in which temperature affects a focal species remains difficult to determine (Anderson et al., 2001; Gilman, 2017; Harvey and Moore, 2017; Murrell and Barton, 2017; Sanford, 2002). Nonetheless, our study shows that clearance rate increased linearly with temperature, but also provides evidence for the indirect roles of temperature on food resources and zooid states that could affect clearance rates (Fig. 4). An important next step will be to link clearance rates to ingestion (i.e. filtration rate; Riisgård, 2001), and to understand whether clearance rates meet the metabolic demands under higher temperature, and how clearance rate affects fitness. For example, if clearance rates reflect respiration, then elevated respiration can reflect a beneficial capacity to support work, or the detrimental consequence of rapid consumption of energy reserves. Another important next step will be to test whether and how flow speed alters the effect of temperature on clearance rate. *Bugula neritina* is a suspension feeder that actively draws food particles to its mouth using its cilia-lined lophophore tentacles, such that water flow around the colonies can affect their ability to obtain food. At this stage, it is unclear how flow speed would interact with the effect of temperature on clearance rate in active suspension feeders.
